# Clinical presentation, predictive factors and management of patients with Nelson syndrome: a retrospective study

**DOI:** 10.1007/s11102-025-01579-0

**Published:** 2025-09-26

**Authors:** Pierluigi Mazzeo, Giulia Bovo, Alessandro Mondin, Giacomo Voltan, Renzo Manara, Mario Caccese, Luca Denaro, Filippo Ceccato, Mattia Barbot

**Affiliations:** 1 Department of Medicine-DIMED, University of Padova, Padua, Italy; 2 Endocrinology Unit, University-Hospital of Padova, Via Ospedale Civile 105, Padua, 35128 Italy; 3 Neuroradiology Unit, University-Hospital of Padova, Padua, Italy; 4 Department of Neuroscience, University of Padova, Padua, Italy; 5 Department of Oncology, Veneto Institute of Oncology IOV- IRCCS, Oncology 1, Padua, Italy; 6 Academic Neurosurgery, Department of Neurosciences, University of Padova, Padua, 35121 Italy

**Keywords:** Cushing disease, Corticotrophinoma, Nelson syndrome, Bilateral adrenalectomy, Target therapy

## Abstract

**Background:**

Nelson syndrome (NS), or corticotroph tumor progression after bilateral adrenalectomy (CTP-BADX/NS), is a serious complication in patients with Cushing disease (CD) following BADX. Surgical tumor removal is the recommended treatment, though adjuvant therapies may be necessary.

**Aim of the study:**

To evaluate clinical, radiological, and hormonal features of CD patients after BADX, identify risk factors for CTP-BADX/NS and assessed treatment outcome and cardio-metabolic complications.

**Methods:**

Retrospective study of 30 patients (male/female: 9/21; median age at CD diagnosis: 33 years, IQR 27–42) who underwent BADX and had a minimum follow-up of 18 months. Data were collected at diagnosis and during follow-up (6, 24 months and last visit).

**Results:**

Over a median follow-up of 135 months, 9/30 patients (30%) developed NS, median 60 months after BADX. NS patients had earlier CD diagnosis and higher ACTH levels two years post-BADX [458 ng/L (IQR 245–723) vs. 146 ng/L (61–247), *p* = 0.020]. They also took lower fludrocortisone [0.05 mg/day vs. 0.1 mg/day, *p* = 0.001] and tended to use less hydrocortisone [20 mg/day [20–25] vs. 30 [25–30], *p* = 0.06]. Pre-BADX stereotactic radiosurgery (SRS) was more frequent in non-NS patients (52% vs. 22%, *p* = 0.11).

Hypertension was more common in NS patients (78% vs 43%), but diabetes less so (33% vs 48%). In the CTP-BADX group, 6/9 required pituitary surgery and/or radiotherapy; medical therapy was used in 5 patients with varied results.

**Conclusion:**

CTP-BADX/NS occurred in 30% of cases in our cohort. Higher ACTH post-BADX and younger age at CD onset may predict NS. No hormonal or radiological markers reliably predicted tumor progression. SRS before BADX and higher hydrocortisone doses might offer protection. Tumor control often needed a multimodal approach, with limited success from medical therapy alone.

**Supplementary Information:**

The online version contains supplementary material available at 10.1007/s11102-025-01579-0.

## Introduction

Cushing disease (CD) caused by an adrenocorticotropic hormone (ACTH)-secreting pituitary adenoma is the most common etiology of endogenous Cushing syndrome [1]; persistently elevated ACTH levels lead to excessive cortisol secretion which is associated with increased morbidity and mortality compared to the general population [2]. The treatment of choice for CD is transsphenoidal surgery (TSS), aiming to selectively remove the adenoma; however, a significant number of patients require second-line treatments due to incomplete tumor removal or disease recurrence following surgical remission [1–3]. Surgical failure depends on tumor volume, its invasiveness and surgeon’s level of experience. Recurrent disease may develop even several years after surgery, although the highest risk is observed within the first five years following TSS. Therefore, lifelong surveillance is essential in these patients [3–5].

Second-line treatments for persistent and recurrent CD include repeat TSS, fractionated pituitary irradiation or radiosurgery, medical therapy targeting pituitary tumor, adrenal cortisol production or glucocorticoid receptors and, lastly, bilateral adrenalectomy (BADX) [1, 2]. Resorting to bilateral adrenalectomy has significantly decreased in recent years thanks to the development of new drugs effective in managing hypercortisolism; however, it remains indicated in certain specific cases [1, 2].

In fact, BADX is a highly effective treatment that may be considered in young women seeking pregnancy or when other therapies are ineffective or unsuitable, particularly in patients with severe disease [1, 2, 6].

However, BADX is generally considered a salvage therapy for CD, since it results in permanent adrenal insufficiency requiring lifelong steroid replacement therapy and carrying the risk of life-threatening adrenal crisis; additionally, there is a continuous risk of ACTH-secreting tumor regrowth due to the loss of negative feedback from cortisol.

Nelson Syndrome (NS) is a possible complication of BADX in patients with resistant CD; historically, when first described it was defined by three criteria: progression of a pituitary ACTH-secreting tumor, skin hyperpigmentation and high ACTH levels [7].

In 2021, an expert consensus was reached following a comprehensive review of the literature on NS, resulting in updated recommendations [8]. The first recommendation focused on the terminology of the syndrome, suggesting that the term NS should have been replaced with “progression of the corticotropic tumor after bilateral adrenalectomy/NS” (CTP-BADX/NS) [8, 9]; this change aimed to emphasize that the central feature of the condition is tumor progression.

Secondly, the diagnostic criteria for CTP-BADX/NS were redefined, with the main criterion being radiological evidence of tumor progression or the detection of a new radiologically visible pituitary tumor after BADX. In contrast, the presence of skin hyperpigmentation or a progressive rise in plasma ACTH levels after BADX were considered non-mandatory secondary criteria for diagnosis [8].

However, to date, there are limited data on potential predictive factors for the development of NS after BADX and, since NS is a rare condition, data on the optimal management of these patients are even more scant.

## Patients and methods

We conducted a monocentric, retrospective study involving 30 patients with CD who underwent BADX due to refractory disease [female 21 (70%), male 9 (30%), median age at CD diagnosis 33 years, IQR 27–42] with a minimum follow-up of 18 months after adrenal surgery, followed at the Endocrinology Unit of the University Hospital of Padova between January 1986 and May 2025.

The primary aims of our study were to evaluate clinical, radiological and hormonal characteristics of patients with CD submitted to BADX and to identify potential risk factors for the development of CTP-BADX/NS.

Secondary objectives were to describe treatments used to prevent tumor progression and the onset of NS, while assessing their effectiveness in real-world clinical practice.

All patients were diagnosed with hypercortisolism based on clinical features and confirmed through appropriate hormonal testing, including low-dose dexamethasone suppression test, 24-hour urinary free cortisol (UFC), and late-night salivary cortisol (LNSC) measurements [10].

The diagnosis of ACTH-dependent syndrome due to ACTH-secreting pituitary adenoma was confirmed on the strength of detectable ACTH levels > 10 ng/L, the pathological response of second line tests and/or the inferior petrosal sinus sampling when pituitary MRI and dynamic testing were not conclusive and, lastly, with the histological examination in those submitted to TSS [10–13].

We retrospectively collected data on clinical, biochemical, and radiological findings, as well as treatments administered to control cortisol secretion including surgery, radiotherapy, and medical therapy, and the development of cortisol-related comorbidities such as arterial hypertension, impaired glucose homeostasis, dyslipidemia, overweight, and osteoporosis.

Moreover, we evaluated metabolic and hormonal parameters at 6 months, 2 years, and at the last available visit after BADX.

Lastly, we collected data on treatments used to control excessive cortisol secretion in CD prior to BADX, as well as therapies applied to manage tumor regrowth in cases of CTP-BADX/NS.

CTP-BADX/NS was defined as radiological evidence of progression of an existing adenoma after BADX or the new occurrence of a pituitary tumor in patients in whom no adenoma was visible on previous MRI. Tumor progression was considered significant with an increase of at least 2 mm in one of the three dimensions of the adenoma together with at least one of the three following features: increase in pituitary stalk deviation; enlargement of the upward convexity of the diaphragma sellae; increase asymmetry of the sellar floor [9].

This study was conducted in accordance with the principles of the Declaration of Helsinki and received approval by the Ethical Committee of the Province of Padua (PITACORA, protocol number AOP3318, Ethic Committee registration 5938-AO-24).

### Statistical analysis

Statistical analyses were performed using GraphPad Prism (GraphPad Software Inc., San Diego, CA, USA) and a p-value of less than 0.05 was considered statistically significant.

Categorical variables were reported as frequencies or percentages, while quantitative variables were expressed as medians with interquartile ranges (IQR).

Comparisons between dependent groups were made using the Wilcoxon test for non-parametric quantitative variables, while the Mann-Whitney test was used to compare non-parametric quantitative variables between independent groups.

For the nominal variables, the χ2 test and Exact Fisher’s Test, when there were small samples, were used for independent samples.

## Results

During the observation period (median time 135 months, IQR 105–362), 9/30 patients (30%) developed NS after a median time of 60 months (IQR 33–108) from BADX (Table 1).

**Table 1:** Clinical, radiological and therapeutic treatments of the whole CD cohort. Continuous variables are expressed as median and interquartile range (IQR)

Parameters	Only BADX (*n* = 21) median (IQR)	CTP-BADX/NS (*n* = 9) median (IQR)	GLOBAL (*n* = 30) median (IQR)	*P* value BADX vs. CTP-BADX/NS
**- **Sex, n (%) - Female - Male	15 (71%) 6 (29%)	6 (67%) 3 (33%)	21 (70%) 9 (30%)	NS (0.99) NS (0.99)
**-** Survival at last visit, n (%)	16/21 (76%)	7/9 (78%)	21/30 (70%)	NS (0.99)
**- **Follow-up after BADX, months	135 (40–370)	150 (110–341)	135 (105–362)	NS (0.99)
**- **Age at CD diagnosis, years	34 (28–46)	30 (26–33)	33 (27–42)	NS (0.14)
**-** Age at last visit, years	61 (45–69)	55 (48–68)	60 (47–68)	NS (0.72)
**-** Type of adenoma ** -** negative/NA, n (%) ** - **microadenoma, n (%) ** - **macroadenoma, n (%)	4 (19%) 14 (67%) 3 (14%)	2 (22%) 5 (56%) 2 (22%)	6 (20%) 16 (53%) 5 (17%)	NS (0.82) / / /
**- **TSS, n (%)	17/19* (89%)	8/9 (89%)	24 (80%)	NS (> 0.99)
**- **Time from diagnosis to TSS, months	7.5 (5–13)	8 (6–24)	7 (5–13)	NS (0.78)
**- **SRS/RT before BADX, n (%)	11/19* (52%)	2/9 (22%)	13 (43%)	NS (0.11)
**-** Medical treatment for CD, n (%)	13/19* (68%)	7/9 (78%)	20/29* (71%)	NS (0.99)
- no treatments ** -** cabergoline - ketoconazole - metyrapone - pasireotide - mitotane	6 (32%) 1 (5%) 12 (63%) 1 (5%) 2 (11%) 0	2 (22%) 2 (22%) 4 (44%) 1 (11%) 1 (11%) 1 (11%)	8 (26%) 3 (10%) 16 (55%) 2 (7%) 3 (10%) 1 (3%)	
**- **Time from diagnosis to BADX, months	41 (22–108)	72 (70–85)	48 (23–105)	NS (0.80)
- Hydrocortisone equivalent dose, mg/day	30 (25–30)	20 (20–25)	30 (20–30)	NS (0.06)
**-** Fludrocortisone dose, mg/day	0.05 (0.05–0.1)	0.1 (0.1–0.1)	0.1 (0.05–0.1)	0.001
**-** BMI, Kg/m^2^ (before BADX)	26.2 (21-28.5)	24.9 (24.0-30.8)	25.8 (22-28.5)	NS (0.94)
**- **Comorbidities (last evaluation) - Hypertension, n (%)	9 (43%)	7 (78%)	16 (53%)	NS (0.12)
**- **Diabetes, n (%)	10 (48%)^	3 (33%)^	13 (43%)	NS (0.69)
**- **Cardiovascular events, n (%)	8 (38%)	2 (22%)	10 (33%)	NS (0.67)
**-** Osteoporosis/fracture, n (%)	5/17 (29%)	5 (56%)	10 (33%)	NS (0.23)

* In 2 patients there were no available data

^ 1 patient developed diabetes only after initiating pasireotide therapy

In our cohort, all patients underwent BADX due to poor control of CD; 25/30 patients (83%) had at least one TSS prior to BADX (median time 7 months, IQR 5–13), one patient received only radiotherapy before BADX, while the remaining 4 patients directly undergone adrenal surgery.

### Clinical, radiological and therapeutic features of the whole cohort

Among all patients, there was a slight difference in age at CD diagnosis between those who developed CTP-BADX/NS and those who did not [30 years (IQR 26–33) vs. 34 years (IQR 28–46), *p* = 0.14] (Table 1).

Interestingly, patients who did not develop CTP-BADX/NS were on lower doses of fludrocortisone [0.05 mg/day (IQR 0.05–0.1) vs. 0.1 mg/day (IQR 0.1–1.12), *p* = 0.001], and we reported a tendency to receive more hydrocortisone [30 mg/day (IQR 25–30) vs. 20 mg/day (IQR 20–25), *p* = 0.06].

Moreover, there were no differences in the time from CD diagnosis to TSS or in the use of medical therapies to manage cortisol excess; however, we noticed a higher proportion of patients submitted to stereotactic radiosurgery (SRS) or conventional radiotherapy (RT) in the group who did not develop CTP-BADX/NS [11/19 (52%) vs. 2/9 (22%), *p* = 0.11], even if the correlation was not significant, probably related to the small sample size (Table 1).

Regarding comorbidities, in the CTP-BADX/NS group 78% of patients (7/9) had hypertension, and 33% (3/9) developed diabetes mellitus; instead, in the group of patients without NS, only 43% of patients (9/21) had hypertension, and similarly 48% developed diabetes.

### Hormonal parameters at diagnosis and follow-up of the whole cohort

We did not observe any difference in baseline hormone values at time of CD diagnosis, except among second line tests, in which in the CTP-BADX/NS group there was a tendency to a lower ACTH increase after desmopressin stimulation test (DDAVP; median 33 ng/L vs. 87 ng/L, *p* = 0.07), while higher ACTH levels after stimulation with CRH (227 ng/L vs. 59 ng/L, *p* = 0.21) (Table 2).

**Table 2:** Hormonal parameters at diagnosis and follow-up of the whole CD cohort. Continuous variables are expressed as median and interquartile range (IQR)

Parameters	Only BADX (*n* = 21) median (IQR)	CTP-BADX/NS (*n* = 9) median (IQR)	GLOBAL (*n* = 30) median (IQR)	*P* value BADX vs. NS
- *ACTH at diagnosis of CD, ng/L	41 (27–76)	30 (27–33)	43 (27–63)	NS (0.69)
- *ACTH before TSS, ng/dL	80 (55–112)	46 (31–52)	80 (57–111)	NS (0.60)
**-** UFC at diagnosis of CD, (x ULN)	2.8 (2.1–4.3)	2.3 (1.4–2.8)	2.7 (2.0-3.7)	NS (0.16)
**-** HDDST response, *n* = 21 - no suppression, n (%) - suppression < 50%, n (%) - suppression > 50%, n (%)	6/15 (40%) 4/15 (27%) 5/15 (33%)	2/6 (33%) 2/6 (33%) 2/6 (33%)	8/21 (38%) 6/21 (29%) 7/21 (33%)	NS (0.94) / / /
**- **CRH test, *n* = 26 - ACTH increment, % - *ACTH increment, ng/L - Cortisol increment, % - Cortisol increment, nmol/L	59 (5.6–216) 47 (4-118) 48 (11–99) 221 (102–406)	227 (186–244) 49 (30–52) 77 (44–104) 301 (185–383)	97 (7.4–233) 48 (4.7–113) 58 (18–102) 221 (113–394)	NS (0.21) NS (0.32) NS (0.61) NS (0.88)
**-** Desmopressin test, *n* = 13 - ACTH increment, % - *ACTH increment, ng/L - Cortisol increment, % - Cortisol increment, nmol/L	273 (153–513) 87 (56–171) 49 (31.5–78.6) 426 (207–665)	94 (71–195) 33 (23–50) 24 (19.6–43) 149 (130–221)	216 (116–400) 67 (44–145) 41 (28–85) 320 (166–596)	NS (0.16) NS (0.07) NS (0.23) NS (0.16)
**-** *ACTH after BADX, ng/L - at 6 months - at 2 years - last available visit before NS	170 (79–246) 146 (61–247) 80 (39–230)	227 (176–553) 458 (245–723) 618 (364–1628)	215 (137–267) 201 (81–413) 175 (43–425)	NS (0.12) 0.020 0.028

*ACTH range values: 4.7-48.8 ng/L; ULN, upper limit of normal

However, we detected significantly higher ACTH levels 2 years after BADX in the CTP-BADX/NS group [458 ng/L (IQR 245–723) vs. 146 ng/L (IQR 61–247), *p* = 0.020]; this difference persisted and further increased alongside the duration of follow-up.

### Clinical parameters and therapeutic options in the CTP-BADX/NS group

In the CTP-BADX/NS cohort, 6 patients (67%) required pituitary surgery (median time 108 months after NS development) and/or SRS to manage tumor growth (Table 3**)**.

**Table 3:** Clinical parameters and therapeutic options in the CTP-BADX/NS cohort. Continuous variables are expressed as median and interquartile range (IQR)

Parameters	Value median (IQR)
**-** Female/Male, n (%Female)	6/3 (66%)
- Time BADX to CTP-BADX/NS, months	60 (33–108)
- Follow-up after CTP-BADX/NS, months	86 (71–276)
- Type of adenoma - negative, n (%) - microadenoma, n (%) - macroadenoma, n (%)	2 (22%) 5 (56%) 2 (22%)
- Tumor progression after BADX, mm	6 (2–33)
- Tumor progression after BADX, %	58 (40–220)
- Hyperpigmentation, n (%)	5 (56%)
- TSS after CTP-BADX/NS, n (%)	5 (56%)
- Time from CTP-BADX/NS to TSS, months	108 (41–170)
- SRS/RT after CTP-BADX/NS, n (%)	7 (78%)
- TSS + SRS/RT, n (%)	5 (56%)
- Medical Treatment, n (%) - no treatment - cabergoline - temozolomide - other combinations (see Table 4)	5 (56%) 4 2 1 2
- Disease stability, n (%)	7 (78%)

Seven patients (78%) obtained disease stability during the follow-up period, and severe tumor progression after NS diagnosis occurred in three patients.

Medical therapy was globally attempted in 5 cases, with huge heterogeneity regarding type of medication and the efficacy on tumor progression (Table 4). Three patients with a severe tumor enlargement were treated with temozolomide (TMZ), of whom one patient discontinued TMZ after four years maintaining disease stability in the follow-up period. The other two patients, briefly discussed below, received combined treatment and initially achieved disease stability; however, both eventually experienced disease progression that caused their death.

**Table 4:** Management of the CTP-BADX/NS cohort

Patient *n*	Age at last evaluation, years	Glucocorticoid treatment after BADX, mg/day	TSS after NS (*n*)	Tumor volume, mm	Ki-67 (%)	SRS/RT after NS	ACTH at NS diagnosis, ng/L	Type of medical treatment	ACTH last evaluation, ng/L	Duration (months) of follow-up after CTP-BADX/NS
**1**	42	Cortisone acetate 25 + 12,5	Yes (1)	11	< 3%	Yes	1850	Cabergoline 3 mg/week for 5 years before TSS and RCH	867	75 - ongoing
**2**	58	Hydrocortisone 10 + 10	Yes (2)	37	NA	Yes	249	Cabergoline 3.5 mg/week - Pasireotide LAR 60 mg + Temozolomide	9726	356 - *death*
**3**	59	Hydrocortisone 10 + 10	Yes (2)	25	NA	Yes	NA	Temozolomide 4 years	2863	321 - ongoing
**4**	72	Cortisone acetate 18,75 + 6,25	No	5	NA	No	438	/	139	157 - ongoing
**5**	48	Dual-Release Hydrocortisone 20 + 5	Yes (2)	48	8%	Yes (3)	3080	Cabergoline - Temozolomide + Pasireotide LAR/Octreotide - Bevacizumab - Everolimus	12,790	86 – *death*
**6**	51	Dual-Release Hydrocortisone 20 + 5	No	NA	NA	No	980	Cabergoline 1 mg/week	430	78 - ongoing
**7**	45	Cortisone acetate 25 + 12,5	No	18	NA	Yes	2485	/	1082	52 - ongoing
**8**	68	Hydrocortisone 10 + 5 + 5	Yes (1)	5	NA	Yes	NA	/	48	280 - ongoing
**9**	68	Cortisone acetate 18,75 + 6,25	No	7	NA	Yes	581	/	404	46 - ongoing

### Case report 1 (Fig. 1)

A 17-year old male (patient n 2) diagnosed with CD underwent TSS in 1982, followed by a second TSS in 1988 and conventional radiotherapy in 1991 due to tumor progression. He subsequently underwent BADX in 1998 and was diagnosed with CTP-BADX/NS in 1999, based on significant tumor growth. In light of this, further TSS procedures were performed in 1999 and 2005 due to persistent tumor growth. Subsequently, adjuvant treatment with cabergoline (1 mg/week) was initiated. The tumor remained stable until 2009, when a marked increase in both ACTH levels and tumor volume was observed. Cabergoline was increased to 2.5 mg/week, leading to partial biochemical control. The patient was then switched to pasireotide LAR (60 mg), resulting in further reductions in ACTH levels and temporary stabilization of tumor growth. Despite stable ACTH levels during follow-up, progressive tumor enlargement continued over the years, with a notable increase in 2019. At that point, temozolomide (TMZ) was started at a dose of 240 mg/day (orally, 5 days per 28-day cycle) in combination with pasireotide LAR, and administered for ten cycles. An initial tumor response was observed; however, progression recurred two years after TMZ discontinuation. The patient ultimately died due to worsening of his clinical condition.

**Fig. 1:**
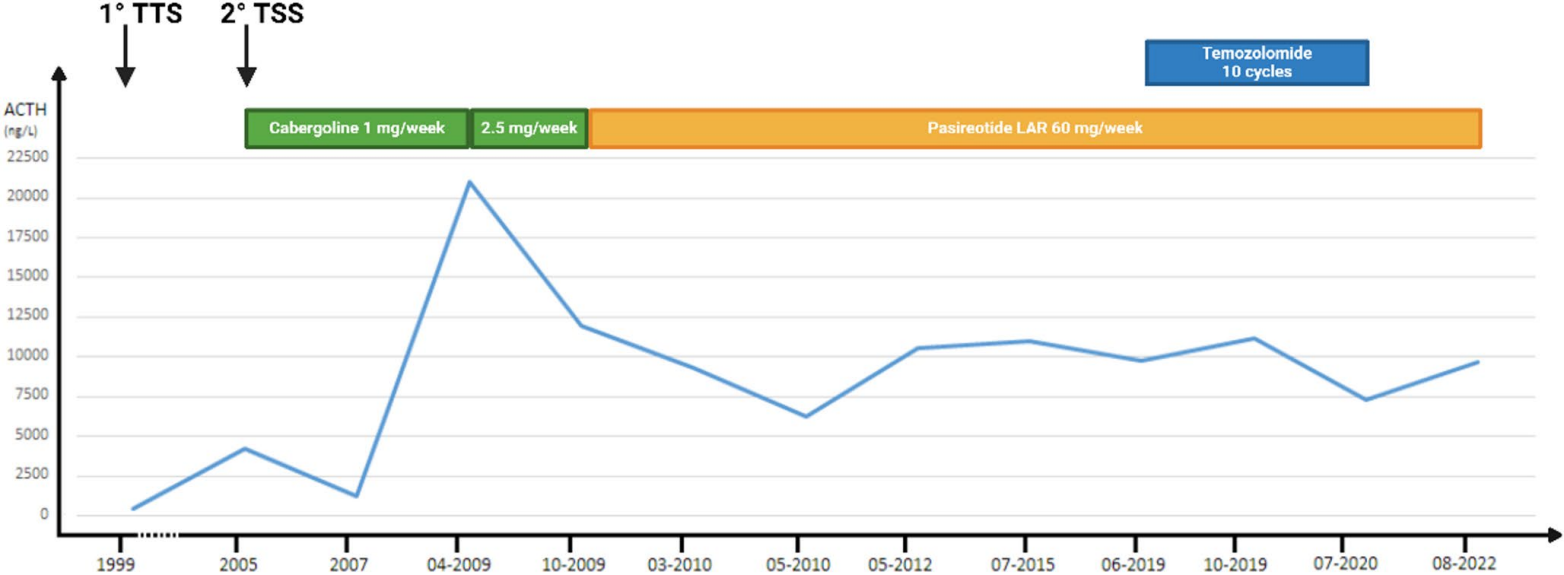
Timeline of the management of patient n. 2. *Created with Biorender*

### Case report 2 (Fig. 2)

A 33-year old female (patient n 5) was diagnosed with CD in 2007 and underwent TSS a few months later; however, due to persistent disease, she was treated with cabergoline and ketoconazole and subsequently received SRS in 2011. In 2013, BADX was performed due to poor disease control. In January 2015, CTP-BADX/NS was diagnosed based on significant tumor enlargement and a marked increase in ACTH levels (3080 ng/dL). Initially, the patient declined surgery, thus medical therapy with cabergoline was initiated and gradually titrated to 3 mg/week, combined with another SRS. After SRS, cabergoline was discontinued. Over the next three years, ACTH levels remained stable, but a progressive tumor growth was observed (Fig. 3). Cabergoline was subsequently reintroduced as a bridging therapy while waiting for repeat TSS. Histopathological analysis confirmed a densely granulated corticotroph pituitary neuroendocrine tumor (Pit-NET) infiltrating the sphenoidal sinus mucosa and adjacent bone, with a Ki-67 proliferation index of 8%. Following a substantial rise in ACTH levels and the onset of visual field defects, TMZ was initiated at 200 mg/m²/day orally for 5 days every 28 days. Pasireotide LAR (40 mg monthly) was added later due to high somatostatin receptor expression seen on 68Ga-DOTATOC PET imaging. This combination led to resolution of visual symptoms, marked ACTH reduction and significant tumor shrinkage on MRI (from 45 × 28 × 21 mm to 30 × 16 × 13 mm) (Fig. 3, panel **D**). Unfortunately, severe hematologic toxicity, including medullary aplasia and neutropenia, necessitated TMZ dose reduction to a metronomic schedule and eventual discontinuation after five cycles. Pasireotide treatment was maintained. Twelve months following discontinuation of TMZ, the radiological evidence of tumor progression was documented. In June 2022, a second therapeutic attempt was initiated using low-dose metronomic TMZ (40 mg/day) in combination with a third SRS. Despite an initial reduction in tumor volume, disease progression recurred within a few months. Following the identification of a Y489C missense mutation in the NF1 gene through next-generation sequencing (NGS), the patient was treated with bevacizumab (two cycles) followed by everolimus (four cycles). Unfortunately, due to clinical deterioration, all therapies were eventually discontinued and palliative care was provided.

**Fig. 2:**
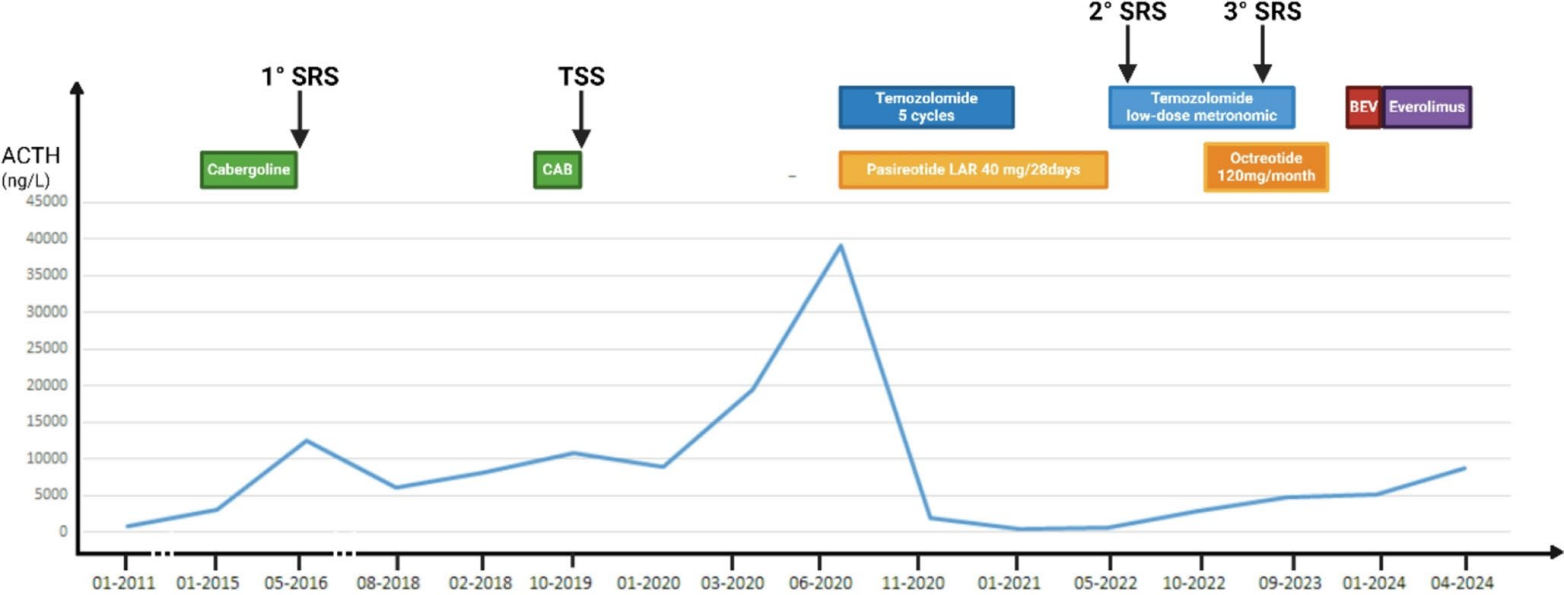
Timeline of the complex management of patient n. 5. Created with Biorender

**Fig. 3:**
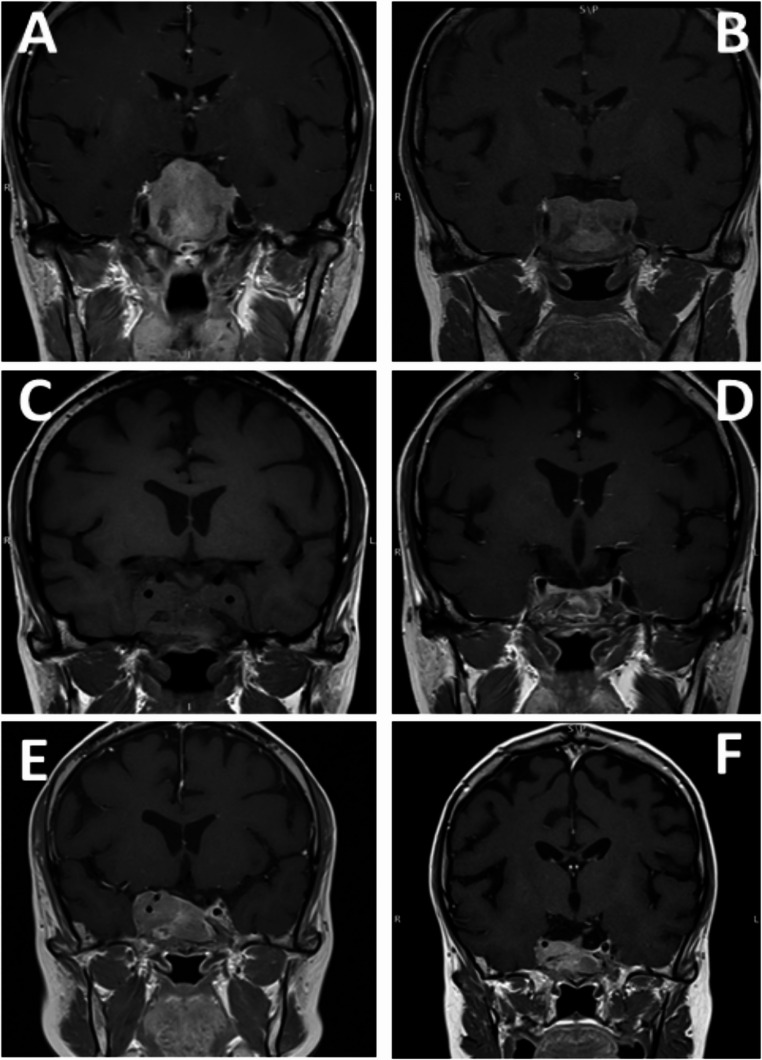
Magnetic resonance images of patient n. 5 Magnetic resonance imaging (MRI) in T1 weighted sequences of patient n. 5 showing tumor lesion after BADX prior to TSS (Panel** A**); tumor debulking after TSS (Panel **B**); tumor re-growth 12 months post-TSS and prior to medical treatment (Panel **C**). Tumor shrinkage following treatment with TMZ plus pasireotide (Panel **D**). MRI 12 months after discontinuation of TMZ, showing tumor progression with invasion of the clivus and right cavernous sinus, along with initial narrowing of the carotid siphon (Panel **E**). Partial tumor regression after a second course of TMZ combined with SRS (Panel** F**)

## Discussion

In the last decades, management of CD has evolved significantly, with advancements in both diagnostic tools and therapeutic approaches; previously, diagnosis of CD was relied heavily on clinical presentation and biochemical tests, with lower ability of radiological imaging to detect small lesions; on the contrary, in the past years the advance in MRI technique and the use of stronger magnetic field increased the capability to detect tumor progression and pituitary lesions in whom no adenoma was previously visible, thus possibly enhancing also the detection rate of CTP-BADX/NS [14, 15].

However, CTP-BADX/NS is nowadays a relatively rare complication, as advancements in medical treatments and surgery have improved control of cortisol excess, thus reducing the need for BADX, which has become a last-resort option for CD patients [1].

For instance, Gamma Knife SRS has been employed since the mid-20th century to treat recurrent CD. Compared to conventional RT, it delivers highly focused, high-dose radiation, thereby minimizing damage to surrounding healthy tissue while enhancing the effectiveness of disease control [16, 17].

Medical treatment itself has evolved over the past years, with a growing emphasis on targeted therapies and novel steroidogenesis inhibitor like osilodrostat, becoming the cornerstone of management of patients with CD also after the failure of primary surgery [1].

In our cohort, we retrospectively analyzed patients with CD followed at our center over a period of approximately 40 years. Among them, only 30 patients underwent bilateral adrenalectomy (BADX), with a relatively even distribution over the decades (see Supplementary Table 1). Of these, 30% (9/30) developed CTP-BADX/NS, a frequency consistent with previously reported data in the literature [18–20].

Indeed, in a recent meta-analysis which considered thirty-six studies, with a total of 1316 CD patients treated with BADX, the prevalence of NS ranges between 5% and 53.4%, with a pooled prevalence of 26% [21].

Regarding possible precocious predictive factors of CTP-BADX/NS development, our results partially confirmed data in the literature. High ACTH levels after BADX seem to be the most reliable predictor of CTP-BADX/NS [9, 22–24]; some authors proposed that in the first year after BADX, the absolute ACTH variation between consecutive MRIs of more than 100 ng/L gave the strongest association to CTP [9], while other studies defined a cut-off of ACTH levels greater than 300 ng/L after BADX as independent predictors of NS development [25].

Younger age at CD diagnosis or at BADX was also positively associated with the occurrence of CTP-BADX/NS [8, 9, 26–29], since an earlier disease onset may reflect a more aggressive corticotroph tumor phenotype, which has a higher likelihood of progression or recurrence over time. For these reasons, some authors recommend that patients younger than 35 years should undergo closer surveillance, particularly in the first years after BADX [28].

Regarding other predictive factors of CTP-BADX occurrence at the time of CD diagnosis, the presence of a detectable pituitary adenoma, especially larger tumor volume at diagnosis predisposes to a higher risk of CTP-BADX/NS after BADX [9, 23, 25–27]. This risk appears particularly relevant in patients with macroadenomas and cavernous sinus involvement [30]. In a large cohort of 47 patients who developed NS, the median tumor diameter was significantly higher in CTP-BADX group (6 mm vs. 1 mm) when hypercortisolism was first diagnosed [26].

Furthermore, higher UFC levels at CD diagnosis have been correlated with an increased risk of CTP-BADX/NS occurrence, although there are conflicting data in the literature. Some studies have highlighted that higher UFC levels at CD diagnosis were associated with NS development, considering UFC as a potential marker of tumor functionality [24, 27, 29]; however, other studies with similar sample sizes, including our own, have found no such correlation [9, 23, 26]. Therefore, this association remains inconclusive and should be interpreted with caution until further evidence becomes available.

The disruption of the hypothalamic-pituitary axis feedback mechanism, resulting from the absence of endogenous cortisol production, has been hypothesized to play a role in CTP-BADX/NS development. The inadequate glucocorticoid replacement therapy, either due to low dosing or poor compliance, following BADX has been identified as an additional risk factor for CTP-BADX/NS, likely due to the improper feedback of the hypothalamic-pituitary axis [29].

We observed that patients in the CTP-BADX/NS group received higher doses of fludrocortisone following BADX, while being treated with lower hydrocortisone-equivalent doses. However, these glucocorticoid doses were within the range considered adequate for the management of primary adrenal insufficiency [31].

The importance of replacement therapy is further emphasized by reports of patients with Addison disease who develop corticotroph pituitary tumors, in whom prolonged hypoadrenalism may have stimulated pituitary enlargement and corticotroph tumor development [32, 33]. However, the absence of corticotroph hyperplasia in the non-tumorous adenohypophysis, along with the rarity of such finding (only 14 cases reported), does not provide strong evidence for a direct causal relationship between corticotroph axis homeostasis and the development of CTP-BADX/NS. Clinically, this suggests that while careful hormonal monitoring remains essential, other factors beyond simple disruption of cortisol feedback likely contribute to tumor progression.

Some authors tried also to evaluate the role of histological and molecular features; for instance, it was assessed that neither the presence of mitoses nor high percentage of Ki67-immunopositive nuclei in tissue sample after pituitary surgery were related to corticotroph tumor progression, even if the small number of patients evaluated in the study could have affected those findings [9]. Moreover, somatic mutations in the ubiquitin-specific protease 8 (USP8), a gene implicated in the pathogenesis of CD [34, 35], have also been evaluated as possible markers of CTP-BADX/NS. USP8 somatic mutations were more commonly found in NS tumors and associated with a poorer biochemical outcome after surgery [36]. However, since these mutations had a comparable prevalence within the whole CD cohort, authors concluded that USP8 mutations are unlikely a potential driver of corticotroph tumor progression. Unfortunately, we are unable to draw conclusions on histological and molecular factors, as such analyses were not systematically performed in our cohort.

Lastly, it has been suggested that prophylactic pituitary irradiation, using either SRS or conventional RT before BADX may be the only effective measure to decrease the risk of developing CTP-BADX/NS or at least delay its onset, regardless of CD severity [25, 26, 29]. In fact, only few studies did not find a significant correlation between pituitary irradiation before BADX and the risk of CTP-BADX onset, but were primarily biased by small sample sizes and the lack of a control group of patients who did not receive pituitary irradiation [23, 27]. Therefore, prophylactic irradiation of the pituitary adenoma remnant and/or sellar region might be considered prior to proceeding with BADX to reduce CTP-BADX occurrence.

Despite advances in the treatment of CD, the management of CTP-BADX/NS remains a challenging clinical scenario. Although most cases can be addressed with surgery and radiotherapy, the potential development of aggressive lesions continues to be associated with significantly increased morbidity [1, 8].

In those patients, medical treatment is typically considered as a second-tier option when surgery or RT/SRS are no longer feasible [8, 37]. To date, several approaches have been evaluated in CTP-BADX/NS patients, with limited and conflicting data reported on their effectiveness in controlling ACTH levels and tumor growth [8].

TMZ, an alkylating agent, is usually prescribed for aggressive pituitary tumors, with different levels of effectiveness [38]. Also in CTP-BADX/NS patients TMZ is used with varying degrees of efficacy [8, 38–41], since it could lead to tumor stabilization or regression to various extent [41]. In some cases, despite an initial reduction in tumor volume, significant regrowth has been observed, typically occurring around six months after completing chemotherapy cycles [38, 41].

In our cohort, we also obtained disease stability with initial tumor shrinkage in patients during TMZ, and in two patients after TMZ discontinuation or dose-reduction we observed tumor progression which was fatal for both patients.

Low activity of O6-Methylguanine-DNA methyl-transferase (MGMT), was associated with a positive outcome in patients with aggressive pituitary adenomas treated with TMZ; so, it was postulated that the assessment of MGMT expression in tissue sample could be also useful in patients with NS to predict the future response to TMZ [42]. However, regardless of MGMT methylation status, which is not routinely performed, a 3-month TMZ trial is generally considered worthwhile for all patients eligible for TMZ [43].

Some authors also evaluated the use of TMZ combined with capecitabine (CAPTEM); CAPTEM is commonly used in neuroendocrine tumors, and few anecdotal successful cases have been reported treating NS [44, 45]; however, capecitabine could be associated with poor compliance due to possible severe hematologic adverse events, and its role still needs to be determined in NS patients.

Moreover, the association of TMZ and targeted therapies, as anti-VEGF factors (bevacizumab) or mTOR inhibitors (everolimus) were used in aggressive pituitary carcinomas, even if with limited data and efficacy [46–49], but to date no cases have been described in patients with CTP-BADX/NS.

We reported the first case of CTP-BADX/NS patient treated sequentially with bevacizumab and everolimus, although this treatment was associated with tumor progression. Importantly, these treatments were not combined with TMZ, since TMZ had previously been discontinued due to severe hematologic adverse effects during the first cycles, and later due to tumor progression following a second attempt at lower doses (Table 4, patient n. 5; **Fig**,** 2**).

Pituitary-targeting drugs have also been used to control tumor growth; some studies reported efficacy of cabergoline in CTP-BADX/NS, particularly after prior pituitary irradiation [50–53]. However, to date there are no predictive factors of responsiveness to cabergoline in NS patients, since the available data are based upon studies with small sample sizes and biases in patient selection.

In addition, SSAs were also tried as possible treatments in these patients. First-generation SSA octreotide, both in daily subcutaneous or long-acting formulation, demonstrated to lower ACTH levels and control tumor growth in some studies [54, 55]. The efficacy of pasireotide, a second-generation SSA, seems controversially in CTP-BADX/NS patients, since, the use of pasireotide was associated with lowering of ACTH levels but a marginal impact on tumor volume [56–58]; also in our patients during pasireotide we observed and initial tumor shrinkage, but over the long-term, despite stabilization of ACTH levels, there was a progressive increase in tumor volume. According to some authors, an acute response to a test dose or the presence of USP8 mutations, similarly as seen in CD, may be favorable predictors of responsiveness to pasireotide also in CTP-BADX/NS patients [56, 59].

## Conclusions

By reviewing the natural history and behavior in our cohort of patients with CD submitted to BADX, we confirmed the global incidence of CTP-BADX/NS previously reported in other studies; moreover, we highlighted the importance of post-operative ACTH monitoring, identifying ACTH increase after BADX as a major risk factor for tumor re-growth; in addition, a greater ACTH response to CRH stimulation test and a blunt response to desmopressin test at CD diagnosis could to be linked to an increased risk of CTP-BADX/NS development.

In the management of NS, surgery has a pivotal role but often a multimodal strategy including SRS and/or medical therapy is required to control more aggressive tumors. The available treatments still play a marginal role especially in monotherapy; however, in our experience, they may contribute to slow down tumor progression.

However, the wide spectrum of CTP-BADX/NS behavior and the lack of prognostic factors, pinpoint the need of a tailored and multimodal approach in these patients.

Further data are essential to explore additional radiological and biological markers, which could improve the prediction of tumor aggressiveness and resistance to medical treatments.

### Limitations

The primary limitations of this study are the small sample size and its single-center retrospective design; while this approach generally ensures consistency in treatment strategies and evaluation of comorbidities, it may introduce bias in patient selection. We also acknowledge the extended observation period as a potential limitation of our study, given that improvements in diagnostic techniques and treatment modalities over time may have influenced disease outcomes and data consistency. However, it should be noted that the rarity of the disease and the need for long-term follow-up for disease onset make prospective evaluation rather difficult.

Further prospective studies are needed to confirm our findings.

## Supplementary Information

Below is the link to the electronic supplementary material.

Supplementary Material 1 (DOCX 20.2 KB)

## Data Availability

All data underlying the results are available as part of the article and no additional source data is required.

## Abbreviations

ACTH: adrenocorticotropic hormone
BADX: bilateral adrenalectomy
CD: Cushing disease
CTP-BADX: corticotroph tumor progression after bilateral adrenalectomy
DDAVP: desmopressin
IQR: interquartile range
LAR: long acting release
LNSC: late-night salivary cortisol
MGMG: O6-Methylguanine-DNA methyl-transferase
NS: Nelson syndrome
RT: radiotherapy
SRS: stereotactic radiosurgery
TMZ: temozolomide
TSS: transsphenoidal surgery
UFC: urinary free cortisol
USP8: ubiquitin-specific protease 8
