# Supplementary material for: Clinical presentation, predictive factors and management of patients with Nelson syndrome: a retrospective study
Source: Pituitary. 2025 Sep 26;28(5):102. doi: 10.1007/s11102-025-01579-0 (PMC12474698; doi:10.1007/s11102-025-01579-0)
Supplement: Supplementary file 1 — Supplementary Material 1 (DOCX 20.2 KB) [file 11102_2025_1579_MOESM1_ESM.docx]

| **Patient n** | Sex | Age at CD diagnosis (years) | TSS before BADX (n) | Conventional RT before BADX | SRS before BADX | Medical treatment  (n of drugs) | TSS after NS (n) | SRS/RT  after NS (n) | Global follow-up period | Year of BADX |
| --- | --- | --- | --- | --- | --- | --- | --- | --- | --- | --- |
| **CTP-BADX/NS** | **Cohort** |  |  |  |  |  |  |  |  |  |
| **1** | Female | 18 | Yes (2) | No | No | Yes (1) | Yes (1) | Yes | 1997-2021 | 2010 |
| **2** | Male | 17 | Yes (1) | No | No | Yes (1) | Yes (2) | Yes | 1982-2020 | 1988 |
| **3** | Male | 30 | Yes (1) | No | Yes | No | Yes (2) | Yes | 1993-2022 | 1994 |
| **4** | Female | 38 | Yes (1) | No | No | Yes (1) | No | No | 1988-2025 | 1995 |
| **5** | Female | 33 | Yes (1) | No | Yes | Yes (2) | Yes (2) | Yes (3) | 2007-2022 | 2012 |
| **6** | Female | 33 | Yes (2) | No | No | Yes (1) | No | No | 2005-2025 | 2013 |
| **7** | Female | 29 | Yes (2) | No | No | Yes (1) | No | Yes | 1986-2001 | 1992 |
| **8** | Male | 27 | No | No | No | No | Yes (1) | Yes | 1982-2025 | 1992 |
| **9** | Female | 49 | Yes (2) | No | No | Yes (2) | No | Yes | 2006-2025 | 2012 |
| **Non NS Cohort** |  |  |  |  |  |  |  |  |  |  |
| **10** | Female | 34 | Yes (1) | No | Yes | Yes (1) | / | / | 1979-2022 | 1988 |
| **11** | Female | 46 | Yes (3) | No | No | No | / | / | 2002-2025 | 2016 |
| **12** | Female | 59 | Yes (2) | No | Yes | Yes (1) | / | / | 1999-2011 | 2010 |
| **13** | Female | 27 | Yes (2) | No | Yes | Yes (1) | / | / | 2004-2022 | 2012 |
| **14** | Female | 46 | No | No | No | Yes (1) | / | / | 1989-2002 | 1990 |
| **15** | Female | 54 | No | Yes | No | Yes (1) | / | / | 1986-1992 | 1986 |
| **16** | Male | 32 | Yes (1) | Yes | No | No | / | / | 1987-2019 | 2004 |
| **17** | Female | 35 | Yes (1) | No | No | No | / | / | 1991-2024 | 2013 |
| **18** | Female | 31 | Yes (1) | Yes | No | No | / | / | 1982-2020 | 1986 |
| **19** | Female | 47 | Yes (1) | No | No | Yes (1) | / | / | 1986-2009 | 1986 |
| **20** | Female | 37 | Yes (2) | No | No | No | / | / | 2007-2012 | 2008 |
| **21** | Male | 18 | Yes (1) | Yes | Yes | Yes (1) | / | / | 1984-2019 | 1988 |
| **22** | Female | 25 | Yes (1) | Yes | No | No | / | / | 1976-2020 | 1989 |
| **23** | Female | 53 | No | No | No | Yes (1) | / | / | 2008-2025 | 2009 |
| **24** | Female | 33 | Yes (1) | Yes | No | Yes (1) | / | / | 1988-1993 | 1991 |
| **25** | Female | 36 | Yes (1) | No | No | Yes (1) | / | / | 1987-2020 | 1989 |
| **26** | Male | 51 | Yes (1) | No | No | Yes (2) | / | / | 2010-2020 | 2012 |
| **27** | Male | 15 | Yes (1) | No | No | NA | / | / | 1992-2021 | 1992 |
| **28** | Male | 28 | Yes (2) | Yes | No | Yes (3) | / | / | 2015-2025 | 2016 |
| **29** | Male | 28 | No | No | No | NA | / | / | 1975-2024 | 1986 |
| **30** | Female | 28 | Yes (2) | No | No | Yes (1) | / | / | 2015-2025 | 2023 |

BADX, bilateral adrenalectomy; CD, Cushing Disease; NS, Nelson Syndrome; RT, radiotherapy; SRS, stereotactic radiosurgery; TSS, transsphenoidal surgery.
